# Can the development of cooking skills influence nutritional status and diet in healthy adults? A systematic review and meta-analysis protocol

**DOI:** 10.1371/journal.pone.0325947

**Published:** 2025-06-13

**Authors:** Eva Débora de Oliveira Andrade, Érika Paula Silva Freitas, Daniele de Souza Marinho do Nascimento, Manuela Mika Jomori, Thais Souza Passos, Grasiela Piuvezam, Bruna Leal Lima Maciel

**Affiliations:** 1 Graduate Program in Health Sciences, Health Sciences Center, Federal University of Rio Grande do Norte, Natal, Rio Grande do Norte, Brazil; 2 Graduate Program in Nutrition, Health Sciences Center, Federal University of Rio Grande do Norte, Natal, Rio Grande do Norte, Brazil; 3 Department of Nutrition, Federal University of Santa Catarina, Florianopolis, Santa Catarina, Brazil; 4 Department of Nutrition, Health Sciences Center, Federal University of Rio Grande do Norte, Natal, Rio Grande do Norte, Brazil; 5 Public Health Graduate Program, Health Sciences Center, Federal University of Rio Grande do Norte, Natal, Rio Grande do Norte, Brazil; 6 Department of Public Health, Health Sciences Center, Federal! University of Rio Grande do Norte, Natal, Rio Grande do Norte, Brazil; University of Sao Paulo, BRAZIL

## Abstract

Cooking skills are defined as the individual knowledge, confidence, and attitude required to perform culinary tasks, which have been studied as a strategy to improve diet and health outcomes. This article describes a systematic review and meta-analysis protocol aiming to assess whether whether intervention-based development of cooking skills improves dietary quality or nutritional status in healthy adults. The protocol follows the guidelines of the Preferred Reporting Items for Systematic Reviews and Meta-Analyses Protocols [PRISMA-P] and has been registered in the International Prospective Register of Systematic Reviews database (PROSPERO: CRD42022385234). Search strategies will be developed based on the combination of descriptors and executed in the PubMed, Embase, Scopus, Web of Science, and SciELO databases. Only intervention studies linking cooking skills to dietary outcomes will be included. Two trained researchers will independently select articles, extract data, and assess the risk of bias. The methodological quality of the studies will be evaluated using the Cochrane Risk Of Bias In Non-randomized Studies – of Interventions [ROBINS-I] and the Cochrane Risk-of-Bias tool for randomized trials (RoB 2). The Grading of Recommendations Assessment, Development, and Evaluation (GRADE) framework will be employed to assess the quality of evidence. Implementing this protocol will result in the development of a systematic review assessing whether the enhancement of cooking skills can influence the nutritional status and diet of healthy adults. Systematizing this knowledge is important to understanding cooking skills not only as a therapeutic tool when pathologies are present but also as a health prevention strategy. Therefore, the systematic review based on this protocol may contribute to the formulation of effective public health policies aimed at improving nutritional status and diets through the development of cooking skills, thus helping prevent adverse health outcomes such as cardiovascular diseases, diabetes, and obesity.

## Introduction

Cooking skills are defined as the individual knowledge, confidence and attitude required to carry out culinary tasks [1,2]. These skills involve not only the knowledge necessary for cooking but also include actions such as meal planning, making healthier food choices, purchasing food within the available budget, understanding techniques for food utilization to reduce waste, and optimizing the time required for meal preparation and cooking [3].

High levels of cooking skills consequently can promote better food choices and healthier habits [2,4], especially when these skills involve the use of greater amounts of fresh or minimally processed foods in meal preparation, leading to an increased intake of fruits and vegetables, for example [1,5]. This, in turn, enhances the consumption of fiber, micronutrients, and unsaturated fats [6]. Conversely, these practices result in lower usage and consumption of processed and ultra-processed foods, thereby reducing the intake of saturated fats and sugary foods [4–6].

Excessive consumption of processed and ultra-processed foods is associated with adverse changes in individuals’ general health status, reflected in weight gain and in the development of non-communicable chronic diseases [7–9]. Existing literature reports a negative association between cooking frequency and culinary skills with the reliance on ready-to-eat meals, supporting the cooking-diet-weight relationship [4,6,8].

Despite the recognized importance of culinary skills, few studies have explored their influence on the nutritional status and diet of healthy adults, particularly from a health prevention perspective. The available studies did not adequately systematize the information, nor did they conduct the pertinent analyses of risk of bias, an essential step for understanding the quality of the evidence. Furthermore, they did not aim to perform a meta-analysis of the data presented [10–13]. Therefore, a systematic review and meta-analysis aiming to evaluate whether the development of culinary skills influences the diet of healthy adults is warranted. This article aimed to detail the protocol for a systematic review and meta-analysis, with the aim to determine whether intervention-based development of cooking skills improves dietary quality or nutritional status in healthy adults.

## Methods/design

### Protocol registration

This systematic review protocol was developed following the recommendations of the Preferred Reporting Items for Systematic Reviews and Meta-Analyses Protocols [PRISMA-P] [14]. The protocol has been registered on the Prospective International Systematic Reviews (PROSPERO) platform (registration number: CRD42022385234), available at: https://www.crd.york.ac.uk/prospero/display_record.php?ID=CRD42022385234.

### Review question

The present protocol was designed to address the following question: *Can the development of cooking skills influence nutritional status and diet in healthy adults?* This question was formulated based on the PECOS strategy (P, population; E, exposure; C, comparators; O, outcomes, S, Study design) (Table 1).

**Table 1:** Structure of the research question based on the PECOS strategy and search strategy to retrieve articles for the systematic review and meta-analysis: Can the development of cooking skills influence nutritional status and diet in healthy adults?.

Description	Abbreviation	Elements	Terms
**Population**	P	Healthy adults	healthy adults OR healthy people OR adults
**Exposure**	E	Development of culinary skills	culinary skills OR cooking skills OR cooking education OR food skills OR Cooking OR Food preparation
**Comparators**	C	Healthy adults who have not been exposed to culinary skill interventions	_
**Outcomes**	O	Assessment of nutritional status and/or diet	nutritional status OR body mass index OR diet OR diet quality
**Study design**	S	_	intervention study OR clinical trial

* PECO (P, population; E, exposure; C, comparators; O, Outcomes S, Study design).

### Inclusion and exclusion criteria

This review will include intervention studies published without language restrictions in scientific journals that meet the eligibility criteria and involve a healthy adult population. The eligibility criteria are as follows: intervention studies investigating cooking skills, cooking confidence, culinary and/or nutrition knowledge, nutritional status, diet quality, and dietary habits in healthy adult populations. Regarding the term cooking skills, we will include intervention studies that implemented actions and evaluated cooking skills in terms of knowledge, confidence, and attitude.

This review will include both randomized controlled trials (RCTs) and quasi-experimental or non-randomized studies. The methodological distinctions among these designs will be systematically considered in the assessment of risk of bias, as well as in the stratification of studies into subgroups based on their methodological characteristics. These distinctions will also inform the analytical approach adopted in the synthesis of findings, particularly in the conduct of the proposed meta-analysis. Concerning the assessment of nutritional status, data related to Body Mass Index (BMI) will be considered, as this parameter enables the synthesis and comparison of outcomes across the different studies included in the review.

Systematic reviews, case reports, books, conference proceedings, short communications, editorials, letters to the editor, theses, cross-sectional studies, dissertations, animal studies, and studies involving children and adolescents will be excluded.

### Sources of information and literature search

The systematic review and meta-analysis will be conducted following the protocol registered in PROSPERO, aiming to determine whether the development of cooking skills can influence the nutritional status and diet of healthy adults.

A comprehensive search strategy will be developed using combinations of keywords and Boolean operators (AND and OR) in electronic databases – PubMed, Embase, Scopus, Web of Science, and SciELO, without language restrictions. MESH terms will not be used for equation formulation because “cooking skills”, “culinary skills”, and terms related to the concept of cooking skills are relatively recent terms in the scientific literature that do not have an associated MESH term [1,3].

Additionally, a manual search will be performed to include articles that may not have been retrieved from the databases listed above. The search equation for the systematic review will be defined based on the established topics of three of the items of the PECOS strategy (Table 1). Adjustments may be made to the search strategy, considering the literature review and the characteristics of the electronic databases, and terms may be added or modified as needed. The search will be conducted and finalized in May 2025.

### Study selection

The articles will be uploaded to Rayyan (version 2022) [15], where duplicates will be identified and removed. Following the eligibility criteria, two authors (EA and EF) will perform an initial screening of the studies based on the information contained in their titles and abstracts. Subsequently, a full-text screening of the articles will be conducted by the same independent researchers. A screening of the references of the included articles may also be performed to identify potentially eligible studies that might not have been retrieved in the original database search. The selection or exclusion of studies will be reported using the PRISMA-P flowchart (S1 Fig) in May 2025.

It is worth noting that the team responsible for this work is composed primarily of nutritionists and researchers in the field of Nutrition, which positively contributes to the selection and screening of studies, as this process is carried out by experts with subject-matter expertise.

### Data extraction

After selecting the studies to be included in the review, two reviewers will independently prepare Microsoft Excel spreadsheets containing data from these articles, in May 2025. The following information will be extracted and summarized in the spreadsheets: study characteristics [title, author, year and language of publication, study location, and study design]; population characteristics [health status, sample size, age, and gender of participants]; methodology; description of results; relevant conclusions; and reported limitations (Table 2). All data extracted from included studies will be made publicly available upon publication of the final review.

**Table 2:** Data extraction table template.

Study characteristics	Population characteristics	Methodology	Results	Conclusions	Reported limitations
					

### Data analysis and synthesis

Data will be presented in summary tables and in narrative form to describe the characteristics of the included studies. If the included studies were sufficiently homogeneous [16,17], we will perform a quantitative synthesis of the results in May 2025. The expected completion of the analyses and submission of the final manuscript is July 2025.

For data analysis and synthesis, subgroups will be constructed considering the type and duration of the interventions implemented in the included studies. For the meta-analysis, it is intended to use information on Body Mass Index (BMI) and validated dietary quality assessment instruments available in the articles to be included, with the aim of analyzing the outcomes related to nutritional status and diet. The results obtained in the included studies will be extracted to calculate the delta of variation [o] and standard deviations of variation for the intervention and control groups. Then, in the comparative analyses the results will be presented through the standardized mean differences (SMD) between the groups [intervention and control] for each included study [16,17].

When the studies included in the review present distinct effect measures, such as odds ratios (OR) or raw means, the conversion of these measures to a common metric, such as the standardized mean difference, will be considered whenever this transformation is statistically feasible and methodologically appropriate, considering the appropriate assumptions [18]. In cases where conversion is not possible or reliable, subgroup analyses will be performed, according to the type of effect measure used. For studies with incomplete statistical data, effect sizes will be estimated from information such as p-values, confidence intervals or data extracted from graphs, according to the guidelines of the Cochrane Handbook [19]. When such estimates cannot be performed, the studies will be included only in qualitative synthesis, with justified exclusion from the quantitative meta-analysis.

To perform the meta-analysis, Review Manager 5.4 will be used (The Nordic Cochrane Centre: Copenhagen, Denmark) [20], a software recommended by the Cochrane Handbook for Systematic of Interventions [21]. The random effects model will be used to calculate the total effect size of the studies included in the meta-analysis.

The evaluation of heterogeneity between studies will be verified by chi-square test (X^2^) with a significance level (p < 0.05) and I^2^ statistical tests. I^2^ assesses the proportion of variability between studies and levels that can be classified into low levels (0% to 25%), medium levels (above 25% to 50%), or high levels of heterogeneity (above 50%). If substantial heterogeneity occurs, we will perform subgroup analysis and meta-regression to identify possible associated cofactors by sex, age, and risk of bias. If possible, funnel plots will be used to assess the presence of potential reporting biases. A linear regression approach will be used to evaluate funnel plot asymmetry [16,17].

### Risk of bias

The risk of bias will be assessed by two trained independent reviewers (EA and EF) in May 2025. Non-randomized intervention studies will be reviewed using the Cochrane Risk Of Bias In Non-randomized Studies – of Interventions (ROBINS-I) [22]. Randomized intervention studies will be reviewed using the Cochrane Risk-of-Bias tool for randomized trials (RoB 2) (Table 3) [23].

**Table 3:** Tools for analyzing risk of bias and quality of evidence, types of related studies and classification.

Risk of bias assessment		
**Tool**	**Types of studies**	**Classification**
ROBINS-1	Non-randomized studies	Low risk of bias; Moderate risk of bias; Serious risk of bias; Critical risk of bias; No information [for specific domains, if applicable]
RoB 2	Randomized trials	Low risk of bias; Some concerns; High risk of bias
**Quality of evidence**		
GRADE	Set of evidence from all types of studies	High quality; Moderate quality; Low quality; Very low quality

The Grading of Recommendations, Assessment, Development, and Evaluation (GRADE) framework will be used to assess the level of evidence. GRADE is a systematic approach to rating the certainty of evidence in systematic reviews and other evidence syntheses. GRADE classifies evidence as high (indicating a high degree of certainty that the association is unlikely to change), moderate (indicating moderate certainty that the association may not change), low (indicating low certainty that the association may not change), and very low (indicating very limited certainty about the estimated association, making any findings uncertain) [24].

## Discussion

This protocol aimed to guide a systematic review and meta-analysis that aims to determine whether the development of cooking skills can influence the nutritional status and diet of healthy adults. Intervention articles focusing on the development of cooking skills in their population samples, through theoretical or practical courses on food preparation, nutrition, and nutrition education will be selected.

Cooking skills appear to be related to exposure to culinary practices within the family environment, where greater contact is associated with higher levels of culinary skills [1]. These practices were traditionally passed down from generation to generation and often assigned to women [3,6]. Such skills can improve diet and strengthen healthier eating habits [4,25], involving greater use of fresh or minimally processed foods, leading to an increased intake of fruits and vegetables [1,8], and subsequently increasing fiber, micronutrients, and unsaturated fat consumption [6]. Conversely, cooking skills allow for lower consumption of processed and ultra-processed foods, resulting in reduced intake of saturated fats and sugary foods [4–6].

The literature already highlights the importance of cooking skills for health, serving as a therapeutic tool for improving nutritional status and quality of life in the presence of pathologies, especially non-communicable chronic diseases such as cancer [26–28], diabetes [29–33], cardiovascular diseases [34–38], and obesity [5,8,39]. Several studies also emphasize the influence of cooking skills on the health, diet, and nutritional status of specific populations, such as children [40–44], adolescents [45–49], and pregnant women [50–54]. However, few studies have explored the influence of cooking skills on the health, nutritional status, and diet of healthy adults, particularly from a preventive health perspective. The studies available in the literature that proposed to address this topic have limitations regarding the rigorous and systematic presentation and analysis of data. It is also observed the absence of an assessment of the risk of bias, a fundamental step for the adequate assessment of the quality of the evidence. Furthermore, unlike what is proposed in this protocol, these studies did not perform meta-analysis models for the quantitative synthesis of the findings [10–13].

Among the potential limitations of the studies used to construct the review, some points include the low quality of the original included studies, varying intervention methodologies, and heterogeneous measurement of outcomes across selected studies. Despite these limitations, the present protocol aims to systematically provide evidence on whether the development of cooking skills in healthy adults can influence their nutritional status and diet.

Thus, this protocol will guide the development of the first systematic review and meta-analysis addressing the development of culinary skills and their influence on nutritional status and diet, and consequently on the general health of the healthy adult population. The review’s findings may contribute to understanding the importance of cooking skills not only as a therapeutic tool when pathologies are present, particularly chronic ones, but also as an alternative for health prevention. Consequently, it will serve as a valuable instrument for the development of effective public health policies aimed at improving diet through the development of cooking skills, aiding in the prevention of adverse health outcomes such as cardiovascular diseases, diabetes, and obesity.

## Supporting information

S1 Fig Article selection flowchart adapted from Preferred Reporting Items for Systematic Reviews (PRISMA-P). (DOCX)
