# Supplementary material for: Can the development of cooking skills influence nutritional status and diet in healthy adults? A systematic review and meta-analysis protocol
Source: PLoS One. 2025 Jun 13;20(6):e0325947. doi: 10.1371/journal.pone.0325947 (PMC12165349; doi:10.1371/journal.pone.0325947)
Supplement: S1 Fig — (DOCX) [file pone.0325947.s001.docx]

**Identification of studies via databases and registers**

Records removed *before screening*:

Duplicate records removed (n =)

Records marked as ineligible by automation tools (n =)

Records removed for not evaluating cooking skills (n=)

Records removed due to unhealthy population (n=)

Records removed because they are not adult population (n=)

Records removed for other reasons (n = 6)

Records identified from:

Pubmed (n =)

Embase (n =)

Scopus (n =)

Web of Science (n =)

Scielo.org (n =)

Scileo.br (n =)

**Identification**

Records screened

(n =)

Records excluded**

(n =)

Reports not retrieved

(n =)

Reports sought for retrieval

(n =)

**Screening**

Reports excluded:

Reason 1 (n =)

Reason 2 (n =)

Reports assessed for eligibility

(n =)

Studies included in review

(n =)

**Included**

Fig 1. Article selection flowchart adapted from Preferred Reporting Items for Systematic Reviews (PRISMA-P).
